# Grounded Theory of Barriers and Facilitators to Mandated Implementation of Mental Health Care in the Primary Care Setting

**DOI:** 10.1155/2012/597157

**Published:** 2012-07-29

**Authors:** Justin K. Benzer, Sarah Beehler, Christopher Miller, James F. Burgess, Jennifer L. Sullivan, David C. Mohr, Mark Meterko, Irene E. Cramer

**Affiliations:** ^1^Center for Organization, Leadership, and Management Research, VA Boston Healthcare System, Boston, MA 02130, USA; ^2^Boston University School of Public Health, Boston, MA, USA

## Abstract

*Objective*. There is limited theory regarding the real-world implementation of mental health care in the primary care setting: a type of organizational coordination intervention. The purpose of this study was to develop a theory to conceptualize the potential causes of barriers and facilitators to how local sites responded to this mandated intervention to achieve coordinated mental health care. *Methods*. Data from 65 primary care and mental health staff interviews across 16 sites were analyzed to identify how coordination was perceived one year after an organizational mandate to provide integrated mental health care in the primary care setting. *Results*. Standardized referral procedures and communication practices between primary care and mental health were influenced by the organizational factors of resources, training, and work design, as well as provider-experienced organizational boundaries between primary care and mental health, time pressures, and staff participation. Organizational factors and provider experiences were in turn influenced by leadership. *Conclusions*. Our emergent theory describes how leadership, organizational factors, and provider experiences affect the implementation of a mandated mental health coordination intervention. This framework provides a nuanced understanding of the potential barriers and facilitators to implementing interventions designed to improve coordination between professional groups.

## 1. Introduction

Recent years have seen increasing recognition that the integration of mental health services into the primary care arena is an essential step for improving quality of mental health care [1, 2]. Based on this evidence, the Veterans Health Administration of the Department of Veterans Affairs (VA) has undertaken one of the largest implementations of Primary Care Mental Health Integration (PC/MHI) in the world [3]. The purpose of this integration was to improve the coordinated identification and treatment of prevalent mental health and substance use disorders in the VA primary care context [4].

At its broadest level, PC/MHI involves the implementation of structural organizational changes to policies, procedures, and practices that are intended to promote collaboration between primary care and clinical mental health experts in the assessment, treatment, and management of common mental health conditions [4]. As such, PC/MHI constitutes an organizational intervention to improve the coordination of patient care by effecting changes in care processes. The systematic implementation of PC/MHI across VA began with the first large-scale request for proposals occurring in 2007 [4]. Since then, spread of PC/MHI has been swift: as of 2011, over 85% of the more than 140 VA medical centers nationwide report some level of integration of mental health services into primary care. A 2010 issue of *Families, Systems, and Health* described many aspects of this national mandate within VA [4, 5].

 Along with the rapid spread of PC/MHI throughout VA medical centers, research has investigated the factors important for successful PC/MHI implementation. For example, recent studies have explored the challenges faced by providers and clinics (within and outside VA) in transitioning to a PC/MHI model. These challenges include clarifying the responsibilities of PC/MHI versus specialty mental health clinics [6], balancing the availability of different types of appointments within PC/MHI [7], adjusting the timing and length of PC/MHI sessions [8], and establishing guidelines for smooth coordination between providers [9]. In addition, others have investigated factors that are important to consider when implementing large-scale changes across networks of VA medical centers [10].

 With a few exceptions, PC/MHI research has been conducted in the context of implementations aimed at maintaining fidelity to a specific model or form of PC/MHI [8, 10, 11]. For example, the St. Louis Initiative for Integrated Care Excellence or SLI^2^CE [6] carried an explicit emphasis on the integrated-collaborative-care model (e.g., time-limited interventions for a broad spectrum of disorders). VA, however, did not mandate one way of accomplishing PC/MHI. Instead, VA provided initial funding and allowed local site discretion in the implementation of at least one of three foundational models of integrated care: colocated collaborative care [12] and two models of care management [13, 14]. These models were similar in the introduction of a multidisciplinary treatment team but differed with respect to format (e.g., phone versus in-person), scope (e.g., depression versus broad range of mental health conditions), and types of care emphasized (e.g., triage versus care management versus brief interventions) [5]. The majority of VA facilities implemented some form of colocated care, with some sites including elements of care management, but rarely with strict fidelity to a theoretical model [5]. Thus, the conceptual differences in models may not be as pronounced in the local adaptations of PC/MHI. Despite isolated case studies [7], little is known about locally varied implementation of PC/MHI in response to the 2007 nationwide VA mandate.

 The potential conflict between top-down-planned organizational changes and the autonomy of local practices has long been a concern in integrated health care systems such as VA, but the question of how to balance local needs for adaptation to mandated organizational changes with standardizing pressures is also broadly important to the private sector. For example, the Systems of Care program for children's mental health is similar to PC/MHI in that both are national programs that provide funding and guidelines for integrated care, while granting considerable autonomy to local sites/communities in tailoring the organizational changes [15]. More broadly, as part of the accreditation process, The Joint Commission often mandates certain outcomes and allows sites to determine the appropriate structure and processes for achieving them [16]. In addition, medical home and accountable care organization innovations in the US health care system are creating environments where these kinds of interventions are highly likely to be more common, particularly for psychotherapy interventions that have been poorly translated into community settings and/or limited to specialist mental health clinical settings [17].

 The present paper addresses this gap in knowledge about the ability of local sites to respond to mandated organizational changes regarding mental health coordination processes. Rather than focus on fidelity to a specific model of PC/MHI (e.g., SLI^2^CE), this study extends the scope of extant PC/MHI research by investigating the factors that may affect the ability of local clinical leaders to implement the structures and processes needed to coordinate mental health care across different VA clinics. There are several implementation models that have been used in the VA context [18, 19], but none of these models provide guidance as to how organizational factors (e.g., leadership, communication, staffing, physical space) may affect the ability of local sites to respond to a mandated organizational intervention. The concept of organizational coordination in health care was used to explicate the operational processes in PC/MHI and examine how organizational barriers and facilitators affected the ability of local sites to respond to the PC/MHI mandate.

### 1.1. Organizational Coordination in Integrated Care

The current study used an organizational coordination framework to guide our inquiry. We conceptualized the coordination processes involved in managing interdependent tasks across professional and team/unit boundaries to achieve integrated care. Organizational coordination can be achieved through standardized or interpersonal coordination processes [20, 21]. Standardized coordination processes (e.g., referral systems) are used to manage common coordination needs and interpersonal coordination processes (e.g., communication between providers, curbside consults) are used to manage less predictable coordination needs. Research has shown that both coordination modalities are needed for high-quality care [20]. Organizational coordination theories are well suited to elucidate the relationship between organizational structures, processes of care, and health outcomes [22] because they provide opportunities to identify organizational factors relevant to the PC/MHI implementation without unnecessarily constraining the data to conform to existing organizational models/structures.

Effective integrated care must be clinically appropriate as well as financially and operationally viable [23].The current study only examines the operational factors that influence implementation of coordination procedures. However, we acknowledge that operational factors are only one dimension of integrated care. Regarding the clinical dimension, we conceptualize both patient experiences and quality of care as important clinical outcomes of integrated mental health care. Outcomes of integrated mental health care include clinical outcomes such as increased identification and treatment of mental health symptoms and patient subjective experiences such as perceived access to mental health care and reduced stigma [24], but it is possible for organizational changes intended to promote integrated care to be implemented in ways that serve the needs of providers and administrators more than patients. Regarding the financial dimension, we acknowledge that improvements in quality of care should be cost effective, and thus cost is an important criterion for judging PC/MHI [25]. We conceptualize PC/MHI as an organizational coordination intervention that has the potential to impact access to mental health care, patient experiences of their care, quality of patient care, and healthcare costs.

Regarding the operational dimension, we conceptualized PC/MHI as an organizational coordination intervention because it involves planned changes to the task-based standardized and interpersonal interactions between healthcare staff across team or unit boundaries (i.e., primary care and mental health). These operational changes represent structural integration of services that are exogenous to our framework of PC/MHI. Structural integration has been identified as a mechanism to promote coordination of patient care, but as noted in recent reviews, organizational changes that are intended to integrate services may not translate into either improved patient experiences [26] or effective collaborative care [27]. In order to build theory regarding how structural integration may or may not impact coordinated care, we focused our study on the processes of coordination (i.e., standardized and interpersonal) and allowed the organizational factors to emerge from the interviews. As shown in Table 1, our interview questions and data reflect this conceptual framework.

## 2. Methods

### 2.1. Participants

Key informants included 30 clinic leaders (12 PC physicians, 10 psychologists, 5 psychiatrists, 4 nurses, 3 social workers, 1 physician assistant) who were recruited from 16 PC/MHI clinics across eight VA medical centers as shown in Table 2. Interviewees were recruited from a hospital-based clinic and up to two large outpatient clinics (more than 10,000 unique patients) in each of eight medical centers. Informants varied in their tenure both at VA and also in the current position. Tenure in VA ranged from 1 to 32 years (Mean = 10.03, SD = 7.40). Tenure in current position ranged from 1 to 23 years (Mean = 5.53, SD = 5.07). Leaders were predominantly male (i.e., 12 female, 16 male), whereas staff were predominantly female (23 female, 10 male). The interviews were conducted as part of a managerial evaluation of the implementation of PC/MHI in these medical centers. Institutional review board approval was obtained to report this managerial evaluation data.

### 2.2. Data Collection

Semistructured telephone interviews were conducted between July and August 2009 to measure influences of implementation progress and effectiveness. Stratified purposeful sampling was used by first interviewing both PC and MH leaders who then, respectively, identified primary care and colocated mental health staff. Leaders used their understanding of the local clinic contexts to select professionals representing different clinical backgrounds who could best speak to coordination procedures or who were most closely involved with mental health treatment based on their local context judgment. The purpose of these interviews was to understand the implementation of colocated, collaborative care in local sites. Although some sites also implement depression care management, these models are less common in VA. Interviews required up to 45 minutes with a note taker instructed to record responses verbatim where possible.

In-depth interviews allow for intensive exploration of a phenomenon with individuals who have experienced it [28]. The evaluation team developed seven interview questions and specific probes to gather information on the processes used to coordinate primary care and mental health staff, how those processes changed over time, and how processes could be further improved (Table 1). Questions were generated based on management literature on coordination and were designed so the conversation became progressively more focused on the important aspects of informants' experiences they chose to discuss in the initial open-ended questions. Interviewers first asked two “grand tour” questions [29] that allowed informants to describe the present and evolved/past processes of care used in their sites openly and without a researcher-imposed framework. These questions focused informants on the processes used to provide depression care in the primary care setting and in particular on the changes in those processes. Interviews then turned toward interviewer-driven topics related to characteristics of the intervention (e.g., interpersonal relationships and coordination procedures), and how the intervention could be improved (e.g., communication, collaboration, resource barriers). These follow-up questions allowed us to explore our research questions in more detail [30, 31].

### 2.3. Data Analysis

The data analysis methodology was chosen to elaborate extant theory regarding the role of organization and leadership factors in intervention implementation. Very little theory has been developed regarding how healthcare organizations react to mandated organizational changes; however, we recognized that research on intervention implementation [17, 32–34] and organizational coordination [22] would likely be relevant. We used grounded theory techniques to identify emergent concepts and specify relationships between these theoretical concepts [35]. Specifically, we used NVivo [36] to analyze the data iteratively in four stages.

The purpose of the first two stages was to immerse ourselves in the data. In the first stage, we read through the interviews in their entirety to develop site summaries in order to begin theorizing about the factors that impacted PC/MHI implementation and to understand the gestalt PC/MHI implementation. These site summaries were needed to ensure that coded data were interpreted within the specific context of each site. In the second stage, the first author coded the raw data without any preconceived framework, looking for similarities across our key informants and across our sites. The purpose of this step was to identify similar types of data that could be analyzed in more depth. The output of this step was a set of codes that represented descriptive characteristics of PC/MHI (e.g., types of referral processes).

**Figure 1:**
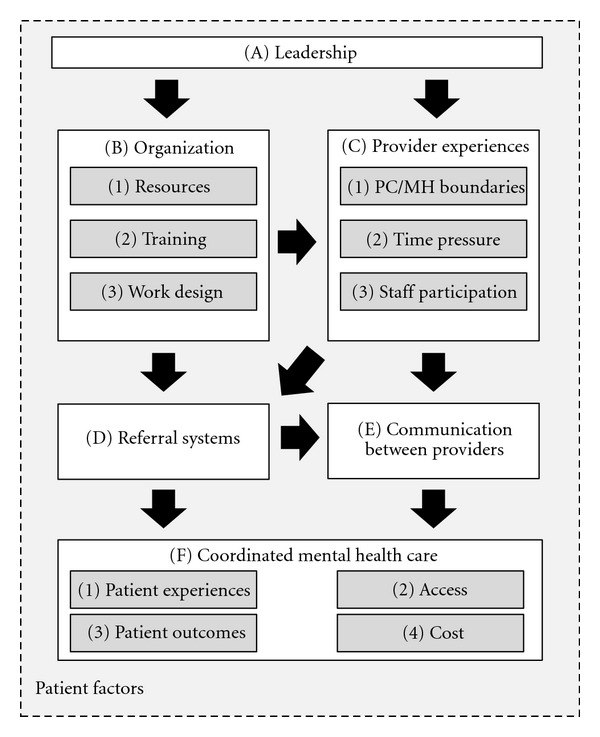
Theoretical framework of barriers and facilitators to locally-adapted PC/MHI implementation.

The purpose of the last two stages was to develop conceptual codes that represented potential causal factors that might impact the PC/MHI implementation (see Table 3) and identify how these codes are connected. Our knowledge of theoretical concepts from implementation science as well as the leadership and organizational psychology literature served as a foundation for our analysis. The concepts and framework presented here, however, were grounded in and emerged from the data analyses [37]. In the third stage, we reanalyzed the raw data associated with each descriptive code. Two coders (one, [blinded], trained in the organization sciences and a second, [blinded], an expert in mental health services) sequentially recoded the evaluation data in NVivo (version 2) to identify potential causal relationships among the codes [38]. Each first coder identified the relevant concepts through an iterative process of coding emergent concepts, reviewing the concepts, revising the conceptual codes, and developing a coding guide for the second coder, who then also coded the emergent concepts. The two coders discussed all disagreements, referred to the overall site summaries as needed and reached consensus for the definitions and application of each code. Thus, the codes represented a consensus between these two disciplinary perspectives. The fourth stage of analysis focused on establishing the connections between the concepts represented in this set of codes. Specifically, the data were divided between four authors such that each author was responsible for the data associated with 4-5 conceptual codes (e.g., one author reviewed only comments in the leadership factor). The authors then individually analyzed their set of data to identify how their conceptual codes were related to the other conceptual codes listed in Table 3. The outcome of this process was a summary of each conceptual code that detailed the empirical support for causal links with other conceptual codes. With input from all other authors the first author then led a process of integrating these analyses to formulate the emergent theory presented in Figure 1. The first author created an initial path diagram that detailed all of the possible links between concepts suggested by the data. The authors refined the model through an iterative process of reviewing the data and group discussion.

Triangulation is valued in qualitative research because parallel perspectives across key informants can increase the credibility of qualitative analyses [39, 40]. We expected variability in how the intervention was implemented across sites because the design of work is path dependent, that is, the way that structure is modified is influenced by history and prior structure [41], and so work practices are likely to vary greatly by site. We also expected variability in the barriers and facilitators to implementation across sites. Therefore, we compared responses across sites to enhance the credibility of results and we specifically noted where conclusions were based on the report of key informants at only one site.

## 3. Results

We have developed a theory to understand that the leadership and organizational factors create barriers and facilitators to a locally adapted intervention designed to provide mental health care in the primary care setting (Figure 1). Definitions of emergent codes are presented in Table 3. Our analyses focused on factors associated with leadership (Factor A), organization (Factor B), and provider experiences (Factor C) as they were reported to impact both referral systems (Factor D) and communication (Factor E). The role of referral systems and communication with coordinated mental health care (Factor F) was not measured, but informed our conceptual framework and study design, and were discussed as important by participants. Below, we begin by discussing the two modes of coordination in PC/MHI, interpersonal communication between providers, and standardized referral systems. We present evidence regarding the influence of provider experiences on communication (Path C→E), and how referral systems are affected by both organizational factors (Path B→D) and provider experiences (Path C→D). We then review the evidence regarding how provider experiences are affected by organizational factors (Path B→C), and discuss how leadership can affect both organizational factors (Path A→B) and provider experiences (Path A→C). Finally, we conceptually discuss the role of patient factors in our model, depicted as the surrounding environment in Figure 1.

### 3.1. Communication between Primary Care and Mental Health

Communication between providers was relational, starting at the front lines. Primary care providers reported interacting with mental health staff on a personal level, building relationships, in order to generate trust and effective communication. At some sites, where the mental health worker was a psychologist rather than a psychiatrist, primary care providers indicated that they would have preferred MD to MD relations with a psychiatrist. Yet reported communication patterns varied. The source of this variation was not clear from the analyses, but may have been due to individual differences in preferences and attitudes.

Primary care providers who discussed communication barriers indicated that time pressures made it difficult to build relationships (Path C2→E). Communication between PC and MH typically increased incrementally during the implementation of PC/MHI as the psychological boundaries between services decreased and staff increased their participation and involvement in integrated mental health care. As mental health staff started to work to address primary care needs, primary care began communicating more with mental health (Path C3→E).

### 3.2. Referral Systems

The VA electronic medical record provides tools and templates so PC and MH providers can better communicate about patients and, when necessary, see patients sooner. Some examples included a consult screen being successful in increasing patient access and referrals; a triage checklist for PC to handle patients in crisis right away; and integrated medication lists to flag medication interactions. The electronic medical record provides information about how PC and MH are coordinating services for a patient through automated notification processes. This electronic communication appeared to facilitate interpersonal communication regarding patient care (Path D→E). Staff reported barriers due to inadequate information in the electronic medical record which included the quick consult screen not being detailed enough to provide needed information, follow-up appointments not being scheduled appropriately by specialists, and lack of certain fields which could provide increased coordination (e.g., the name of the primary mental health provider).

Appropriate staffing and funding to hire new employees were reported as important for referral system effectiveness (Path B1→D). Some sites emphasized the need to hire more MH and PC providers. Staffing limitations were reported as barriers to implementing the desired work design for PC/MHI. For example, staffing limited colocation of psychologists that in turn limited the availability of same day access to mental health care or short-term therapies. Space resources were needed to allow staff to be colocated within the same space (Path B1→B3) and to provide the staff needed to handle the additional mental health consults (Path B1→D). Notably, leaders often reported that available space in primary care limited the amount of mental health staff that could be hired. Other staffing issues which may impact referral system effectiveness were staff turnover, inability to fill vacant positions, and Human Resources delays inhibiting timely hiring.

Referral systems were also reported to be affected by time pressure and training. Referrals through the electronic medical record were seen as more efficient when workload was high (Path C2→E). Other sites reported that a lack of knowledge of the appropriate mental health referral processes limited the effectiveness of electronic referrals. Strategies for increasing referrals mentioned were training primary care staff (Path B2→D) and developing referral templates (i.e., standardizing coordination) for the electronic medical record (Factor D). Primary care staff reported needing additional training for evidence-based mental health treatment. Staff from various sites reported receiving broad training regarding changes in work design (Path B2→B3) and referral processes (Path B2→D) but not necessarily in-depth training for how to handle specific problems (Path B2→F). In one site, staff reported that training was needed for nurses (e.g., dealing with crisis patients during triage) and clerks (e.g., interacting with angry patients) in addition to primary care providers.

Unresolved conflicts between work design and patient mental health needs were identified as barriers to patient access that limited referral system effectiveness and efficiency. Primary care providers noted that formulary restrictions often made it difficult for them to adjust medications for mental health conditions (prescribing authority in VA is often limited by provider specialty) that increased the need for specialty care referrals (Path B3→D). One mental health clinician reported that competing demands to focus improvement efforts on returning Veterans and traumatic brain injury led to a divided focus on primary care integration (Path B3→D). Having an on-site mental health clinician was noted to help alleviate same day appointment concerns through warm hand-offs (Path B1→B3→D), where primary care providers would introduce patients to MH staff, who would then perform a short assessment. However, some primary care participants reported the use of beepers as an alternative method to manage scheduling and same day appointment access (Path B3→D). Mental health at many sites responded to high demand for care by designing work (i.e., templates and protocols) to manage this demand (Path B3→D). For example, at one site, mental health required a patient to be on the maximum medication dosage before being seen. Another site handled high demand for substance abuse care by mandating that mental health conditions be resolved before addressing substance abuse. In some sites, primary care staff reported ad-hoc workarounds to meet the criteria of the template to manage these access restrictions.

The intentional choices regarding how primary care and mental health work are organized (i.e., work design) created the potential for conflicts when coordination procedures were revised. Conflicts between intended improvements to work design with the broader patient care system created unintended consequences in referral systems (Path B3→D). One type of conflict was observed between the design of routine and urgent mental health care. Some sites implemented an electronic scheduling system where open slots are available and patients in need of MH services can be given immediate access, but increasing the number of open appointment slots was reported to decrease access for regularly scheduled patients. A second type of conflict was observed in how referral systems were specified between services. Interservice referrals in VA were specified by formal or informal agreements, and these prior agreements set the context for unintended consequences when PC/MHI was implemented. In two sites, emergency room staff reacted to the addition of PC/MHI staff by attempting to shift responsibility for urgent mental health care to primary care. Other sites reported that specialty mental health staff began restricting access to only patients with the most severe conditions. Yet another site reported difficulties managing referrals to substance abuse for patients who also had mental health conditions.

### 3.3. Organizational Factors Influence Provider Experiences

The knowledge, skills, and abilities of staff were frequently mentioned as an important organizational resource for integrated care. Some examples of key relevant staff characteristics were motivation to work with and care for Veterans in particular, primary care providers who understand psychology, and competent mental health liaison nurses. Interviewees reinforced the need for collaboration among providers with differing levels and types of primary care and mental health skills. Interviews suggested that mutual awareness of collaboration opportunities was sufficient to create interactions. Persistence of colocated mental health staff in developing relationships and learning about how to address mental health needs in primary care was reported to slowly change primary care providers' participation (Path B1→C3). At the point of the interviews, mental health staffing was not yet sufficient to handle the volume of work in many sites (B1→C2). Time pressure was closely related to work design, where several sites reported efforts to redistribute workload across PC and MH (B3→C2).

Mental health staff members were frequently colocated with primary care staff. Colocation was perceived as improving integration, referrals, and clinician accessibility in part because proximity increased familiarity between primary care and mental health staff (B3→C3). Mental health providers reported that informal conversations with primary care providers, such as in hallways or over lunch, were particularly important in engaging primary care providers. Preexisting collaborative relationships between primary care and mental health were reported to facilitate the intervention (Path B3→C3). In sites without these preexisting relationships, mental health providers reported actively seeking opportunities to demonstrate how mental health could help primary care. Mental health staff attendance at monthly primary care meetings was reported to increase primary care participation and engagement (Path B3→C3). Employees noted that during these meetings, discussions about appropriate consults occurred, and leadership could provide a consistent message regarding the importance of coordinated mental health care.

Cultural norms and work patterns differ between PC and MH, so defining the boundaries of patient care responsibilities and dealing with preconceived notions of how patient mental health care should be coordinated are challenges that require on-going upkeep. Deterioration of interactions across PC and MH boundaries that arose over time was repeatedly described as turf wars and sometimes attributed to lack of resources (Path B1→C1). For example, primary care providers were reported to be worried that PC/MHI would increase demand on their scarce resources, and at one site mental health providers were reported to redirect large numbers of patients to primary care for treatment who were previously treated in specialty mental health (e.g., personality disorders). Colocated mental health staff, who worked on the boundary between primary care and mental health, provided detailed information regarding these conflicts. The general theme is that boundary issues are not static but require continuing attention over time so as not to break down as staff and circumstances change.

### 3.4. The Role of Leadership in Integrated Care

PC/MHI implementation required collaboration between service leaders. At a minimum, engagement of both primary care and mental health leaders in a collaborative effort was needed to support the intervention. Clinic leaders indicated that the intervention benefitted from collaboration with leaders in administration, human resources, nursing service, and specialists in the needs of Veterans returning from Iraq and Afghanistan. Leadership was reported to be effective in bridging boundaries between services, resolving conflicts, requesting and allocating resources, and setting responsibilities for new staff.

PC/MHI is designed to act for multiple constituencies (e.g., PC, MH, and patients), and changes to work design and resources through the PC/MHI intervention affected how those constituencies perceived the intervention. In some cases, these issues were resolved by leader involvement and interservice negotiations. Leaders helped clinicians cross boundaries between PC and MH through formal and informal meetings (Path A→C1). In contrast, other leaders maintained or even strengthened these boundaries between PC and MH by acting as an intermediary between services rather than promoting direct interaction. Leaders were particularly important when conflicts arose across these PC/MH boundaries (i.e., turf wars) over responsibility for patients with urgent mental health needs.

Leadership was broadly identified as important in focusing available resources toward PC/MHI goals (Path A→B1). Sites that reported leaders to be supportive indicated that leadership was particularly valuable for staff recruitment and space allocation. Informants who reported poor leadership support reported long delays for necessary resources, such as changes in the referrals systems that would improve communication. Further, leaders at all levels (e.g., service chiefs, nurse managers) were reported to have an important role in developing training programs that provided mental health knowledge, skills, and abilities needed for the intervention (Path A→B2→B1).

Informants also reported that leadership was an important factor in adjusting work design to improve patient access to mental health care (Path A→B3). Leadership was also reported to have some impact on staff participation (Path A→C3). Leaders were reported to have minimal direct influence in the intervention as changes in care required changes to provider behaviors, but active frontline leader engagement was reported as critical in supporting highly motivated providers (e.g., providing co-located space, supporting work redesign) thereby maintaining but not necessarily creating staff participation (Path A→B1→C3).

### 3.5. Patient Factors

Data indicated that patient factors may affect the utilization and effectiveness of referral systems (Factor D), as well as the amount of communication needed between providers (Factor E). Informants reported the importance of differentiating patients with on-going chronic needs who need to see providers in a MH setting versus patients with short-term needs that can be handled in a PC setting. Providers would like to refer some of these complex patients to treatment programs or long-term care facilities but sometimes have difficulty finding the open slots. Regarding the effectiveness of referral systems, staff reported that patients were frequently noncompliant to prescribed treatment and missed appointments. In addition, complex patients require medication coordination that may increase the amount of communication needed between primary care and mental health.

## 4. Discussion

We developed a theory that posits leadership, provider experiences, and organizational factors as key influences on the successful implementation of new mental health coordination practices in real-world healthcare settings. Specifically, factors associated with leadership and organizational characteristics shaped provider experiences, all of which affected the degree to which mental health care was coordinated through referral mechanisms and communication among providers. Organizational factors and provider experiences were important because they set the structural and relational conditions that facilitated and hindered PC/MHI implementation. Though the relationships between individual provider attributes and intervention implementation are often meaningful, our findings suggest that the organizational processes supporting/constraining implementation are extremely important.

Leaders were able to affect provider experiences by providing opportunities for staff to work across professional boundaries, by resolving conflicts, and otherwise supporting staff who were working to implement the intervention. Leaders also were able to modify organizational factors by obtaining and allocating critical resources (e.g., personnel, space), developing training to close staff knowledge or skill shortages, and adjusting work responsibilities to address implementation challenges as they arose. Provider experiences and organizational factors directly impacted how referral systems and interprovider communication were implemented.

### 4.1. Practical Significance

This study is a first step toward an explanatory model of organizational coordination intervention implementation that could be applied to a wide range of mental health coordination problems. For example, in addition to outpatient coordination of depression care, transitions of mental health care between inpatient settings and primary care are important. A recent VA study suggests that transitions of mental health care after general medical hospitalizations are particularly important for patients with mental health conditions [42]. We propose that the implementation of coordination procedures between inpatient medicine and an outpatient mental health service is likely to be affected by the same set of factors (i.e., leadership, organization, and provider) as the PC/MHI intervention.

This study has direct, practical implications for mental health providers and managers who are considering implementing changes to how patient care is coordinated across multiple healthcare providers and especially when those providers operate across organizational boundaries (i.e., professional silos). In the context of local responses to a mandated intervention, both leadership and organizational factors (i.e., resources, training and work design) were identified as potential antecedents for the implementation of new referral systems and patterns of communication across providers. Provider factors (i.e., perceived PC/MHI boundaries, and staff participation in the intervention) were reported to be important but only indirectly affected by leaders. Sites in the current study either took advantage of preexisting collaborative relationships between primary care and mental health or utilized highly engaged key individuals to attenuate the boundaries between services and increase staff participation.

The PC/MHI intervention was designed to cross intraorganizational boundaries in that coordination procedures were implemented across PC and MH units within one healthcare facility. However, organizational boundaries can also be interorganizational. For example, coordination procedures could be implemented between independent fee-for-service PC and MH providers or provider groups. We propose that our model is also relevant for these types of interventions. That is, communication patterns between independent facilities such as these will likely depend on the strength of organizational boundaries, the time pressure experienced by staff, and the degree to which staff choose to participate in the intervention. Without a common health system as in VA, organizational boundaries may be stronger and participation may be more variable, but we suggest that the concepts will be similar. Accountable care organizations and medical homes being created in the private sector, for example, may have environments amenable to these types of interventions. Resources, training, and work design may also vary more across independent units and therefore implementing standardized referral procedures may be more challenging, but the key relationships between these concepts are likely to be the same. Because we expect increased variation in both organizational factors and provider experiences when implementing interventions across independent facilities, we expect that leadership will be even more important. Shared vision for the organizational change across independent facilities is likely to be particularly important when redesigning work, allocating resources, and bridging interorganizational boundaries.

### 4.2. Limitations and Future Research Directions

The dependent variable for our study was coordination, an operational dimension of integrated care, but we acknowledge that the clinical and financial dimensions of integration are important outcomes of PC/MHI. Patient experiences, patient access, patient outcomes, and cost data would be needed to judge whether the intervention was successful or not. Our study was only able to identify the factors that influenced the implementation process. That is, leadership, organization factors, and provider experiences may be important in changing coordination processes, but those processes may have negligible impact on outcomes. Thus, further research on the clinical, financial, and patient-centered impacts of the PC/MHI model in actual practice settings is recommended.

We did not design this study to measure the impact of patient factors on the intervention implementation. Healthcare integration is typically conceptualized as an organizational factor, but patient experiences of integrated care are an important dimension that is often overlooked in both theoretical and empirical work [26]. We acknowledge that patient preferences and mental health diagnoses vary in the types of referrals and amount of communication needed, and therefore patient factors may moderate the impact of leadership, organization factors, and provider experiences. For example, depression may be entirely managed within the primary care setting, particularly if colocated short-term therapy is available, whereas other mental health disorders, such as schizophrenia, may be managed in specialized clinics. Thus, the characteristics of the patient panel involved are likely to have considerable impact on leadership support, resources, and provider experiences. Our study was not designed to identify these links and thus our data is limited regarding how patient factors influence the delivery of mental health care in the primary care setting.

 This study was limited in that sites were all sampled from VA, a healthcare network with unique organizational structures compared to other healthcare providers in the United States. Aspects such as its hierarchical structure and the influence it has on different medical centers, the degree to which it is a closed system, and its size, may limit the transferability of findings to other settings [41]. However, the purpose of the current study was theory building rather than theory testing and therefore purposeful sampling to identify the processes of interest was appropriate rather than random sampling to identify population parameters [43]. Our use of multiple sites strengthens the emergent theory because identified processes are not limited to a single group of healthcare providers. Theory building is an iterative process that involves analysis of multiple cases and additional research is needed to determine whether and how certain dimensions of the current theory are limited to VA. Some reported barriers (e.g., turf wars) may not be as relevant for coordination between fee-for-service facilities, but we propose that the concepts presented are likely to generalize across contexts. Because our theory is based on local responses to a national mandate to provide integrated mental health care, results may be most directly applicable to responses to national or state-level policy changes (e.g., licensure scope, mental health equity) but may also apply to any local organizational changes intended to improve coordination. Although the specific factors may vary, the concepts identified in the current study are common in studies of organizational intervention barriers and facilitators [32–34]. We propose that our framework of leadership, organization factors, and provider experiences is relevant to the implementation of any intervention designed to create coordination procedures.

We acknowledge that clearly specified interventions with stronger evidence may be more easily marketed to clinicians and may be viewed as easier to adopt by local leaders [18], but research has not focused adequately on understanding situations where “evidence-based” interventions are not available or do not fit with local conditions. Intervention implementation research in clinical settings tends to focus on either adoption [18, 19, 34] or spread [44–46] of evidence-based practices. This type of implementation research is designed to identify the factors that maximize implementation fidelity of a standardized intervention that is being promoted as a best practice. However, establishing fidelity to a standardized guideline may not be optimal for all sites [44]. Recent research suggests that interventions that are tailored to be consonant with local norms, resources, and patterns of practice may be more sustainable than standardized interventions [45]. Furthermore, healthcare settings face problems for which there may not be clear evidence-based solutions available [46, 47]. There are many studies of implementation barriers and facilitators but no general framework to explain how these barriers and facilitators affect organizational changes [32–34]. The current study provides a framework of barriers and facilitators that can be used to study local tailoring and adaption of planned organizational changes.

Future research on the implementation of interventions designed to improve coordination would benefit from careful initial measurement of the identified organizational, leadership and provider/cultural/social factors to assess baseline capacities relevant for implementation. Repeated measurements of these and related factors over time would allow for exploration of the extent to which they mediate/moderate key intervention outcomes. For example, organizational boundaries due to social and cognitive differences between professions may interfere with intervention implementation [48]. Leadership was the only factor that we identified as an exogenous variable; that is, our interviews did not indicate that leadership was influenced by organizational or provider factors, but rather that leadership influenced each of those factors. Repeated measurement of leadership behaviors and communication across these boundaries could provide evidence that leadership can improve implementation of a coordination intervention by interpersonal interactions between professions. Future research would benefit from exploring the qualities and practices of leaders who effectively support the boundary spanning activities of providers that are essential to providing coordinated mental health care.

## 5. Conclusions

 We used grounded theory to develop a conceptual framework that identifies leadership, provider experiences, and organization factors as key antecedents to local changes when new coordination practices are implemented in healthcare settings. Our results suggest that current organizational resources, training, and work design along with psychological barriers between units, time pressure, and barriers to staff participation in the new coordination procedures at a site are each important factors to consider before implementing an organizational coordination intervention. Results may apply to any local changes intended to improve organizational coordination.

## Figures and Tables

**Table 1:** Interview questions and specific probes.

Interview question	Specific probes
(1) Imagine that a patient with depression symptoms comes to the clinic. Can you walk me through a typical process of care	Referral process, differences between diagnoses?
(2) How has this process changed over the past 10 years? (or since you arrived in the clinic)?	Recent changes, challenges, failures, leadership support, referrals, interpersonal interactions, physical structure?
(3) Tell me about your sense of the need for coordination between PC and MH.	Examples of good and poor coordination?
(4) How would you change your clinic to better coordinate care?	Communication, collaboration, resource barriers?
(5) Have you or anyone you know had to develop your own coordination procedures to ensure that patients receive the best care?	Work-arounds, ad-hoc coordination procedures?
(6) Can you tell me about the relationship between the people in the PC and MH clinics?	Face to face contact, trust?
(7) In what situations would you say that teamwork is most important?	Coworkers back each other up

**Table 2:** Sampling of key informants across the sixteen sites.

	Leaders	Physician	Psychologist	Psychiatrist	Nurse	Social worker	Physician assistant
Hospital-based clinics							

Site 1	2	1	2				
Site 2	2	2	1				
Site 3	2	1	1				
Site 4	2	1	2				
Site 5	1	1	1				
Site 6	2	1		1			
Site 7	2	1	1				
Site 8	2	1	1				

Large outpatient clinics							

Site 9	2		1		1		
Site 10	2	1		1			
Site 11	2					1	1
Site 12	1				1	1	
Site 13	2	1				1	
Site 14	2			1	1		
Site 15	2			1	1		
Site 16	2	1		1			

Psychologists, psychiatrists, nurses, social workers and the physician assistant were all associated with the PC-MHI clinic. These providers represent 51% of PC/MHI staff at these sites.

**Table 3:** Emergent codes.

Code	Definition
(A) Leadership	Leadership does/does not provide direction, coordinate between different services, obtain needed resources, make timely decisions, communicate with staff.
(B1) Resources (space)	Lack of space includes barriers due to physical structure of facility, includes lack of space and distance barriers.
(B1) Resources (staffing)	Not enough staff available to provide coordinated mental health care.
(B1) Resources (knowledge and skills)	Specific mention of staff knowledge, skills, or abilities. It includes general comments such as “good staff”
(B2) Training	Training for MH procedures, including training of admin personnel
(B3) Work design	Intentional choices regarding how care is provided; description of how tasks are divided between staff and/or clinics including informal systems work systems designed to overcome other barriers, including mandated tasks and same day appointments
(C1) PC/MH boundaries	Perceived physical and/or psychological barriers between primary care and mental health clinics provide barriers to care.
(C2) Time pressure	Overworked staff, working through admin/lunch time
(C3) Staff participation	Staff “buy-in”, perceptions of mutual PC and MH participation, comfort with PC/MH referrals. It includes the use of formal and informal meetings to increase participation.
(D) Referral systems	Processes used to coordinate care may include specific barriers to the referral process. It including the use of electronic medical record, paging systems, checklists.
(E) Communication	Interpersonal communication, communication between PC and MH.
Patient complexity	Challenges due to complicated mental health conditions and/or medical comorbidities; patients have many health needs, including noncompliance issues
